# Supplementary material for: Adapting COVID-19 research infrastructure to capture influenza and RSV alongside SARS-CoV-2 in UK healthcare workers winter 2022/23: Evaluation of the SIREN Winter Pressures pilot study
Source: PLoS One. 2025 Jun 23;20(6):e0310758. doi: 10.1371/journal.pone.0310758 (PMC12184992; doi:10.1371/journal.pone.0310758)
Supplement: S1 File — (PDF) [file pone.0310758.s001.pdf]

S1 File: Participant research experience survey questions

1. The information that I received before taking part prepared me for my experience on the study.
2. I feel I have been kept updated about the research.
3. I know how I will receive the results of the research.
4. I know how to contact someone from the research team if I have any questions or concerns.
5. The researchers have valued my taking part in the research.
6. Research staff have always treated me with courtesy and respect.
7. I would consider taking part in research again.
8. How likely would you be to recommend participating in the SIREN postal pathway over participating at a SIREN research site?
9. Do you wish to share any feedback comparing your experience on the postal pathway over participating at a SIREN research site?
10. What are the positive aspects for you of participation?
11. What could have made the research experience better?
