# Supplementary material for: Adapting COVID-19 research infrastructure to capture influenza and RSV alongside SARS-CoV-2 in UK healthcare workers winter 2022/23: Evaluation of the SIREN Winter Pressures pilot study
Source: PLoS One. 2025 Jun 23;20(6):e0310758. doi: 10.1371/journal.pone.0310758 (PMC12184992; doi:10.1371/journal.pone.0310758)
Supplement: Attachment — Submitted filename: Response to Reviewers.docx [file pone.0310758.s005.docx]

**Response to Reviewers**

Please see below responses to all comments provided by the academic editor and reviewer. Comments from the academic editor and reviewer are in **BOLD** and responses not in bold.

**Journal Requirements:**

**1. When submitting your revision, we need you to address these additional requirements.

Please ensure that your manuscript meets PLOS ONE's style requirements, including those for file naming. The PLOS ONE style templates can be found at**
[**https://journals.plos.org/plosone/s/file?id=wjVg/PLOSOne_formatting_sample_main_body.pdf**](https://eur01.safelinks.protection.outlook.com/?url=https%3A%2F%2Ftrack.editorialmanager.com%2FCL0%2Fhttps%3A%252F%252Fjournals.plos.org%252Fplosone%252Fs%252Ffile%253Fid%3DwjVg%252FPLOSOne_formatting_sample_main_body.pdf%2F1%2F010f019493e6bb98-13e918bf-e5f0-41ec-9b13-0e43563e7bf5-000000%2FQJvjbvNZeMfpSzxeRRexEvFmAE89m0-U6k0TjbgwZBI%3D195&data=05%7C02%7Ckatie.munro%40ukhsa.gov.uk%7Ce67951b26eb04d235c2208dd3bc75da7%7Cee4e14994a354b2ead475f3cf9de8666%7C0%7C0%7C638732450261821647%7CUnknown%7CTWFpbGZsb3d8eyJFbXB0eU1hcGkiOnRydWUsIlYiOiIwLjAuMDAwMCIsIlAiOiJXaW4zMiIsIkFOIjoiTWFpbCIsIldUIjoyfQ%3D%3D%7C0%7C%7C%7C&sdata=6MYk%2FSSrrsV%2B0Tv3Ka1PMrNtFbdNb6%2Bo7lMXlN5BTEI%3D&reserved=0) **and**
[**https://journals.plos.org/plosone/s/file?id=ba62/PLOSOne_formatting_sample_title_authors_affiliations.pdf**](https://eur01.safelinks.protection.outlook.com/?url=https%3A%2F%2Ftrack.editorialmanager.com%2FCL0%2Fhttps%3A%252F%252Fjournals.plos.org%252Fplosone%252Fs%252Ffile%253Fid%3Dba62%252FPLOSOne_formatting_sample_title_authors_affiliations.pdf%2F1%2F010f019493e6bb98-13e918bf-e5f0-41ec-9b13-0e43563e7bf5-000000%2FTD5zf4pGj0QqVjsiPZr9SZYDu5D5-722n8yXeJN73_Y%3D195&data=05%7C02%7Ckatie.munro%40ukhsa.gov.uk%7Ce67951b26eb04d235c2208dd3bc75da7%7Cee4e14994a354b2ead475f3cf9de8666%7C0%7C0%7C638732450261832771%7CUnknown%7CTWFpbGZsb3d8eyJFbXB0eU1hcGkiOnRydWUsIlYiOiIwLjAuMDAwMCIsIlAiOiJXaW4zMiIsIkFOIjoiTWFpbCIsIldUIjoyfQ%3D%3D%7C0%7C%7C%7C&sdata=vlLKIsUPNIcALWh1i9W4v9yqjd%2BeWZJ4umzVThgA%2FOA%3D&reserved=0)

We have reviewed and updated the manuscript to make sure it is written and formatted to the journal’s style requirements.

**2. Thank you for stating the following financial disclosure:
[The SIREN study was funded by the UK Health Security Agency; the UK Department of Health and Social Care with contributions from the governments in Northern Ireland, Wales, and Scotland; the National Institute for Health Research; Health Data Research UK (NIHR200927; HDRUK2022.0322)].

Please state what role the funders took in the study. If the funders had no role, please state: ""The funders had no role in study design, data collection and analysis, decision to publish, or preparation of the manuscript.""
If this statement is not correct you must amend it as needed.

Please include this amended Role of Funder statement in your cover letter; we will change the online submission form on your behalf.**

We have included the below text in our updated covering letter, as a Role of Funder statement:

"The funders had no role in study design, data collection and analysis, decision to publish, or preparation of the manuscript."

**3. In the online submission form, you indicated that Anonymised data will be made available for secondary analysis to trusted researchers upon reasonable request.].

All PLOS journals now require all data underlying the findings described in their manuscript to be freely available to other researchers, either 1. In a public repository, 2. Within the manuscript itself, or 3. Uploaded as supplementary information.

This policy applies to all data except where public deposition would breach compliance with the protocol approved by your research ethics board. If your data cannot be made publicly available for ethical or legal reasons (e.g., public availability would compromise patient privacy), please explain your reasons on resubmission and your exemption request will be escalated for approval.**

We have included two additional supplementary files which contain 1) anonymised responses to the feedback survey, and 2) aggregate data on participants and tests by fortnight.

**4. One of the noted authors is a group or consortium [SIREN study group]. In addition to naming the author group, please list the individual authors and affiliations within this group in the acknowledgments section of your manuscript. Please also indicate clearly a lead author for this group along with a contact email address.**

We have included the individual authors and affiliations of the SIREN study group into the acknowledgments section of the manuscript, clearly indicating the lead author along with their email address.

**5. Please include your full ethics statement in the ‘Methods’ section of your manuscript file. In your statement, please include the full name of the IRB or ethics committee who approved or waived your study, as well as whether or not you obtained informed written or verbal consent. If consent was waived for your study, please include this information in your statement as well.**

We have included an ethics statement in the methods section of the manuscript.

**6. We note that there is identifying data in the Supporting Information file <S2_File.pdf>. Due to the inclusion of these potentially identifying data, we have removed this file from your file inventory. Prior to sharing human research participant data, authors should consult with an ethics committee to ensure data are shared in accordance with participant consent and all applicable local laws.**

**Data sharing should never compromise participant privacy. It is therefore not appropriate to publicly share personally identifiable data on human research participants. The following are examples of data that should not be shared:

-Name, initials, physical address
-Ages more specific than whole numbers
-Internet protocol (IP) address
-Specific dates (birth dates, death dates, examination dates, etc.)
-Contact information such as phone number or email address
-Location data
-ID numbers that seem specific (long numbers, include initials, titled “Hospital ID”) rather than random (small numbers in numerical order)

Data that are not directly identifying may also be inappropriate to share, as in combination they can become identifying. For example, data collected from a small group of participants, vulnerable populations, or private groups should not be shared if they involve indirect identifiers (such as sex, ethnicity, location, etc.) that may risk the identification of study participants.

Additional guidance on preparing raw data for publication can be found in our Data Policy (**[**https://journals.plos.org/plosone/s/data-availability#loc-human-research-participant-data-and-other-sensitive-data**](https://eur01.safelinks.protection.outlook.com/?url=https%3A%2F%2Ftrack.editorialmanager.com%2FCL0%2Fhttps%3A%252F%252Fjournals.plos.org%252Fplosone%252Fs%252Fdata-availability%2523loc-human-research-participant-data-and-other-sensitive-data%2F1%2F010f019493e6bb98-13e918bf-e5f0-41ec-9b13-0e43563e7bf5-000000%2FYXSqdFPZEeV1IzHBIC7dDgQ5OO7TzLBvHeRpgYVua-k%3D195&data=05%7C02%7Ckatie.munro%40ukhsa.gov.uk%7Ce67951b26eb04d235c2208dd3bc75da7%7Cee4e14994a354b2ead475f3cf9de8666%7C0%7C0%7C638732450261844170%7CUnknown%7CTWFpbGZsb3d8eyJFbXB0eU1hcGkiOnRydWUsIlYiOiIwLjAuMDAwMCIsIlAiOiJXaW4zMiIsIkFOIjoiTWFpbCIsIldUIjoyfQ%3D%3D%7C0%7C%7C%7C&sdata=%2F2TMrp8Hsohw5geJkyxK5lL7ozC10en7gXN6NyRtzVQ%3D&reserved=0)**) and in the following article:** [**http://www.bmj.com/content/340/bmj.c181.long**](https://eur01.safelinks.protection.outlook.com/?url=http%3A%2F%2Ftrack.editorialmanager.com%2FCL0%2Fhttp%3A%252F%252Fwww.bmj.com%252Fcontent%252F340%252Fbmj.c181.long%2F1%2F010f019493e6bb98-13e918bf-e5f0-41ec-9b13-0e43563e7bf5-000000%2F0LSJbCpY6DO3AMx-KDI-wY-lnmKHIMQXnEgvz1ejhtE%3D195&data=05%7C02%7Ckatie.munro%40ukhsa.gov.uk%7Ce67951b26eb04d235c2208dd3bc75da7%7Cee4e14994a354b2ead475f3cf9de8666%7C0%7C0%7C638732450261855396%7CUnknown%7CTWFpbGZsb3d8eyJFbXB0eU1hcGkiOnRydWUsIlYiOiIwLjAuMDAwMCIsIlAiOiJXaW4zMiIsIkFOIjoiTWFpbCIsIldUIjoyfQ%3D%3D%7C0%7C%7C%7C&sdata=2GXi0%2BhOYOXDyTnmd772YSklHXusH03meKsh8QfQyhU%3D&reserved=0)**.

Please remove or anonymize all personal information (Names), ensure that the data shared are in accordance with participant consent, and re-upload a fully anonymized data set. Please note that spreadsheet columns with personal information must be removed and not hidden as all hidden columns will appear in the published file.**

Supplementary file 2 contained the list of members for the SIREN study group. As requested, the list of members has now been included in the acknowledgements section of the manuscript instead, and supplementary file 2 now removed from the submission.

**7. Please review your reference list to ensure that it is complete and correct. If you have cited papers that have been retracted, please include the rationale for doing so in the manuscript text, or remove these references and replace them with relevant current references. Any changes to the reference list should be mentioned in the rebuttal letter that accompanies your revised manuscript. If you need to cite a retracted article, indicate the article’s retracted status in the References list and also include a citation and full reference for the retraction notice.**

The reference list has been reviewed to ensure it is complete and correct. Reference 1 has been updated from a pre-printed article to the published version in NIHR Open Research. Website links for references 2 and 3 have been updated, and citation dates for websites have been included in line with style guidance. In-text citations have been formatted with square brackets.

**Reviewers’ comments:

Reviewer #1: Thank you for allowing me to review "Adapting COVID-19 research infrastructure to capture influenza and RSV alongside SARS-CoV-2 in UK healthcare workers winter 2022/23: Evaluation of the SIREN Winter Pressures pilot study".

The paper was a well designed and written evaluation of the Winter Pressures study and with minor changes outlined below should be published.**

**Line 35 – I’d like to know the breakdown of the participants for pathway A vs B – it is good to know that 33 sites used pathway A (for example) but how many people was that? Especially since for pathway C you have to report participant number (as there are no “sites”)**

We have updated the abstract to specify the number of participants in pathways A and B.

**Line 38 – Instead of 6; 4; 5 in the brackets for median number of test (which is I assume is what that means), you could put median 5, range 4-6, unless it is important that pathway A had 6, etc**.

We have kept these results displayed as median number of tests per pathway as we believe it is important to distinguish the median number of tests for each pathway.

**Line 163 – it might be my misunderstanding but does the “without limit” refer to the content of the free text response (how it could be interpreted as written now) or the length (character limit) of the response?**

We have amended the methods section to clarify that the free text response was “without a character limit”.

**Line 186 – qualitative is spelled incorrectly.**

We have corrected this spelling.

**Line 190 – “The research study team then discussed collectively to finalise themes”**

We have expanded on this sentence to read “The research study team then collectively discussed the themes to ensure clarity of wording and agreement on the presentation of the final themes” to make the role of collective discussion clearer.

**Line 217 – I find it a bit confusing that you’ve switched the order of the comparison for the brackets in this section. I think keep site first and postal second in all comparison brackets. Or some other way (than looking at the denominator) to make it clear which numbers belong to which category.**

We have updated the order within the brackets to keep the order consistent throughout the paragraph, and made it clearer which pathway each result corresponds to.

**Table 2 – it is interesting that the site based participants reported medical reasons more than postal participants too – think about adding that to the wording.**

We have updated the text in the results section to also included this difference.

**Table 2 – can you do any statistical comparison to see if the differences between sites are significant?**

We have conducted a Fisher’s Exact Test to compare withdrawal reasons between site and postal participants, for the withdrawal reasons that we have mentioned in the text, and included this in the methods and results.

**Line 228 – it would also be good to see a statistical comparison for the swab return rate.**

We have conducted a chi-squared test to compare swab return rates between pathways, and also to compare the proportion of tests that were multiplex between pathways, and included this in the methods and results.

**Line 240 – There were 33 sites in pathway A. You say that they switched to multiplex at various dates between 28 November to 16 March. Given this is a large proportion of the participants and it impacts on the ability to identify flu and RSV, it would be useful to know how many each week – given there were still a lot of people tested only for SARS-CoV-2 in the last time point in the figure, it seems that quite a lot of the 33 sites didn’t switch until very late. For all we can tell from the text at the moment, 1 might have switched on 28 November and the other 32 on 16 March! (exaggeration as I see median is in December) I’m not sure if this would be best in a table or figure, and if it should be in the body of the paper or supplemental but I know I would like to know!**

We have updated the results to specify that, on retrospective questioning to confirm the date of assay switchover, we only received responses from 13 of out 33 sites. We have therefore decided not to include a table/figure due to the small number of sites we have data for. However, we hope that by including this additional information on the number of responses, alongside the median and range of dates, this provides sufficient information to describe the potential discrepancy between intended and actual start dates for sites in the sub-study.

**Line 365 – did you provide workshop participants with an option to provide feedback privately? Not all people like to speak opinions in front of a group.**

We have added a sentence to specify that we did not give an option for private feedback in the limitations paragraph of the discussion.

**Reviewer #2: This manuscript presents a relevant study on adapting research infrastructure during the winter of 2022/23 to capture multiple respiratory viruses in UK healthcare workers. However, there are several areas that need improvement before it can be considered for publication.**

**1. Lack of clarity and structure: The introduction could do a better job of clearly stating the overall project, research design, and aims of the sub-study. It seems a bit muddled, please make it clear from the start what the key research question and why use this process evaluation. Second, some of the paragraphs are a bit short in length, it would benefit from clearer delineation between sections such as Background, Methods, Results, and Conclusion. This could enhance readability and allow us for easier navigation.**

We have rewritten the introductory paragraph to clarify the overall project, design, and aims of the sub-study from the start (lines 52-58). Two subheadings have been added to clearly indicate the paragraphs relating to background information on the SIREN Winter Pressures 2022/23 sub-study, and the section on the process evaluation of the sub-study (lines 60 and 95). We have included a sentence on why a process evaluation was chosen (lines 99-101). Several smaller paragraphs have been merged into larger sections of text, and then formatting of the headings and sub-headings has been adjusted in line with previously published examples to give clearer delineation between the sections.

**2. Sample representativeness: There's not enough information about whether the recruited participants and sites are representative of the broader population of UK healthcare workers. How were the sites selected? Were there any biases in the recruitment process? here’s less information.**

We have included additional information in the methods section to clarify that recruitment was an “opt-in” process for sites. Additional information on the recruitment process can be found in our published protocol paper, which is also referenced in the manuscript and again below. We have not included any demographics of participants to compare to the broader healthcare workforce in the UK, as we did not feel it was appropriate for the process evaluation. However, we have also submitted a paper analysing the results from this sub-study which includes a comparison of participants to the broader workforce currently under review at PLOS One. A citation for this is included below:

Broad J, Sparkes D, Platt N, Howells A, Foulkes S, Khawam J, et al. Adapting COVID-19 research infrastructure to capture influenza and respiratory syncytial virus alongside SARS-CoV-2 in UK healthcare workers winter 2022/23 and beyond: protocol for a pragmatic sub-study. NIHR Open Research. 2024;4(1). doi: 10.3310/nihropenres.13517.3

Foulkes, S., Munro, K., Sparkes, D., Broad, J., Platt, N., Howells, A., Omolola Akinbami, Jameel Khawam, Joshi, P., Russell, S., Norman, C., Price, L., Corrigan, D., Cole, M., Timeyin, J., Forster, L., Slater, K., Watson, C. H., Andrews, N., & Charlett, A. (2024). Adapting COVID-19 research infrastructure to capture influenza and respiratory syncytial virus alongside SARS-CoV-2 in UK healthcare workers winter 2022/23: Results of a pilot study in the SIREN cohort. *MedRxiv (Cold Spring Harbor Laboratory)*. https://doi.org/10.1101/2024.12.09.24318698

**3. Data collection issues: methods for data collection, particularly for participant feedback, should be elaborated upon. What specific questions were asked in the feedback survey? How was thematic analysis conducted?**

The questionnaire used to collect participant feedback is included in supplementary information, and a sentence has been added in the body of the main text to signpost to this (lines 169-170).

As the same thematic analysis methods were used for both the participant and research team feedback, methods for both have been detailed together in lines 196-206. We have briefly expanded on this section for clarity.

**4. Data analysis: The results section presents numerical data but lacks sufficient statistical analysis. For example, it mentions that the median number of tests per participant was similar across pathways but does not provide statistical tests (e.g., ANOVA or Kruskal-Wallis test) to support this claim. Also Including confidence intervals for key metrics would strengthen the findings by providing a measure of precision**.

We have conducted a chi-squared test to compare swab return rates between pathways, and also to compare the proportion of tests that were multiplex between pathways and included this in the methods and results. We have chosen to include p-values instead of confidence intervals as a metric for comparison. We have not conducted a statistical test for the median, however, the IQRs are included for comparison across pathways.

**5. Informed Consent: More detail on how informed consent was obtained from participants would be beneficial, especially regarding those re-recruited into the sub-study. And the ethical review process and any relevant ethical considerations specific to this study seems missing.**

We have now included the ethics statement in the methods section, and additional information on the recruitment process can be found in our published protocol paper, which is referenced in the manuscript and can be found below:

Broad J, Sparkes D, Platt N, Howells A, Foulkes S, Khawam J, et al. Adapting COVID-19 research infrastructure to capture influenza and respiratory syncytial virus alongside SARS-CoV-2 in UK healthcare workers winter 2022/23 and beyond: protocol for a pragmatic sub-study. NIHR Open Research. 2024;4(1). doi: 10.3310/nihropenres.13517.3

**6. Low survey response rate: With only 15.5% of participants completing the feedback survey, the results might be highly skewed and not reflective of the whole cohort. Please try to either find a way to increase the response rate in future research or be more cautious when interpreting the survey data.**

We have expanded the limitations section of the discussion to clarify that the results should be interpreted cautiously (lines 413-415).

**7. Discussions: the limitation could consider, for instance, how might biases in participant selection or feedback affect the results? Additionally, how does the variation in local laboratory testing impact overall study conclusions?**

We have expanded on how biases in participant selection might affect generalisability in lines 403-408, including how recruitment methods may influence outcomes. Lines 427-434 have been updated to provide greater detail on how feedback biases and data collection methods may influence results.

We have also expanded on how variation in local laboratory testing may impact the data collection, and therefore conclusions, of the Winter Pressures sub-study 2022/23 in lines 355-357. This limitation is further addressed in the paper outlining the pilot results of the sub-study, currently under review at PLOS One. A citation for this is included below:

Foulkes, S., Munro, K., Sparkes, D., Broad, J., Platt, N., Howells, A., Omolola Akinbami, Jameel Khawam, Joshi, P., Russell, S., Norman, C., Price, L., Corrigan, D., Cole, M., Timeyin, J., Forster, L., Slater, K., Watson, C. H., Andrews, N., & Charlett, A. (2024). Adapting COVID-19 research infrastructure to capture influenza and respiratory syncytial virus alongside SARS-CoV-2 in UK healthcare workers winter 2022/23: Results of a pilot study in the SIREN cohort. *MedRxiv (Cold Spring Harbor Laboratory)*. https://doi.org/10.1101/2024.12.09.24318698

**8. Implications of Findings: The authors should elaborate on what the results mean for future public health strategies regarding respiratory virus testing, particularly in light of potential future pandemics.**

A paragraph has been added over lines 391-400 to outline the implications of these findings for how best to conduct respiratory virus testing in a research cohort under pandemic or emerging public health threat scenarios. We have outlined the benefits of a postal model for control, reduced delays, and study responsiveness to rapidly changing circumstances. We have also added a line to the ‘Conclusion’ section (429-431) to be more explicit about the use of a postal model in emergency scenarios.
