# Supplementary material for: Adapting COVID-19 research infrastructure to capture influenza and RSV alongside SARS-CoV-2 in UK healthcare workers winter 2022/23: Evaluation of the SIREN Winter Pressures pilot study
Source: PLoS One. 2025 Jun 23;20(6):e0310758. doi: 10.1371/journal.pone.0310758 (PMC12184992; doi:10.1371/journal.pone.0310758)
Supplement: Attachment — Submitted filename: Response_to_Reviewers_auresp_2.docx [file pone.0310758.s006.docx]

**Response to Reviewers**

Please see below responses to all comments provided by the academic editor and reviewer. Comments from the academic editor and reviewer are in **BOLD** and responses not in bold.

**Journal Requirements:**

**Please review your reference list to ensure that it is complete and correct. If you have cited papers that have been retracted, please include the rationale for doing so in the manuscript text, or remove these references and replace them with relevant current references. Any changes to the reference list should be mentioned in the rebuttal letter that accompanies your revised manuscript. If you need to cite a retracted article, indicate the article’s retracted status in the References list and also include a citation and full reference for the retraction notice.**

Thank you for highlighting this. We have reviewed our references, and believe it is now complete and correct. We have made updates, specifically:

- Reference #2: the source has been corrected to ‘UK Government Cabinet Office’
- Reference #3: the source has been corrected to ‘Australian Government’ and the following text was slightly simplified by removing ‘In Care DoHaA, editor.’
- Reference #4: updated to a new reference
- Reference #5: the source has been corrected to ‘National Academies of Sciences, Engineering, and Medicine; Health and Medicine Division; Board on Health Sciences Policy; Forum on Drug Discovery, Development, and Translation. Virtual Clinical Trials: Challenges and Opportunities: Proceedings of a Workshop.’
- Reference #6: author first names have been replaced with initials
- Reference #7: authors have been expanded on to list six in total
- Reference #8: DOI added
- Reference #10: clarified the editors of the book and publisher
- Reference #13: expanded ‘UKHSA’ to ‘UK Health Security Agency’

We have checked citations for any retracted works and are not currently aware of any.

**Reviewers’ comments:

Reviewer #1: Thank you for addressing the comments. The paper looks great. I particularly like Table 1 - this makes it really clear, and the addition of stats elsewhere in the paper really support the story.

Could the p values be added to Table 2? This would also help with interpretation. At the very least, it would be good to be able to identify the statistically significant comparisons via an asterisk.**

**The formatting of some of the cross references has caused some of the Table references to drop to the next line. This is usually because an "enter" has got caught up in the table legend. This may be cleaned up in desktopping though.**

Thank you for your comments. We have included an additional column in Table 2 with p-values comparing responses between site-based and postal participants for each withdrawal reason.

We have also gone through and updated the table legends to ensure that they do not cause line dropping when referenced in the text. Many thanks for bringing this to our attention.
